# Epidemiology of hepatitis B and C among risk groups in Czechia

**DOI:** 10.1186/s12889-025-23014-6

**Published:** 2025-05-21

**Authors:** Iva Bendlova, Vojtech Simka, Ekaterina Ryzhova, Tereza Schovankova, Ondrej Holy

**Affiliations:** 1 Science and Research Centre, Faculty of Health Sciences, Palacký University Olomouc, Olomouc, Czechia; 2 Department of Epidemiology of Infectious Diseases, National Institute of Public Health, Prague, Czechia

**Keywords:** VHB – viral hepatitis B, VHC – viral hepatitis C, Risk groups, Epidemiology

## Abstract

Hepatitis B (VHB) and C (VHC) are significant global public health issues, particularly for certain risk groups. In the Czech Republic, individuals who inject drugs (IDUs), incarcerated people, and those with high-risk sexual behaviours are especially susceptible. This research investigates the epidemiology, risk factors, and effects of both diseases on these groups. It analyzes the incidence of VHB and VHC in Czechia over the last twenty years in the post-vaccine era, compares their occurrence, and identifies factors influencing their rates. A total of 28,160 VHB and VHC cases reported in the Czech Republic from 2000 to 2021 were analyzed, categorized into acute and chronic forms. Specifically, there were 8,762 cases of VHB and 19,398 cases of VHC. The research employed quantitative methods and descriptive data analysis. A spatial visualization of disease occurrence per 100,000 inhabitants in Czech districts was conducted for the years 2000, 2010, and 2020 to compare disease development and the risk group of IDUs across districts. For VHB, transmission primarily occurs through intravenous drug use or risky sexual behaviour, with IDUs, men who have sex with men, and promiscuous individuals being the most at-risk groups. For VHC, sexual transmission is less common, with IDUs being the most at-risk group. Many VHC cases have been recorded in prisons, often due to shared razors or amateur tattooing and piercing. This discovery underscores the necessity for focused interventions and thorough strategies to address both diseases in high-risk communities.

## Introduction

Viral hepatitis remains a significant global public health challenge, affecting millions of individuals worldwide and contributing to substantial morbidity, mortality, and economic burden. The World Health Organization (WHO) estimates that hepatitis B virus (VHB) and hepatitis C virus (VHC) infections account for approximately 1.1 million deaths annually, primarily due to complications such as cirrhosis and hepatocellular carcinoma [7]. Despite the availability of preventive and therapeutic strategies, hepatitis B and C continue to disproportionately affect vulnerable populations, including people who inject drugs (PWID), men who have sex with men (MSM), incarcerated individuals, and individuals with limited access to healthcare services [5].

The transmission occurs predominantly through parenteral routes, including exposure to contaminated blood and bodily fluids. Common transmission pathways include the use of non-sterile medical equipment, blood transfusions with inadequately screened donor blood, organ transplantation from infected donors, and needle-sharing among PWID. Hepatitis B virus, which is highly infectious, can also be transmitted through sexual contact and perinatal exposure from mother to child, whereas hepatitis C is primarily associated with blood-to-blood contact and is less efficiently transmitted via sexual activity [10, 30]. The infectious dose of VHB is as low as 1–10 viral particles, making it significantly more contagious than VHC, which requires an estimated 10,000 particles for successful transmission [9].

In the Czech Republic, the epidemiology of viral hepatitis has evolved over the past two decades, particularly due to the introduction of preventive measures such as mandatory hepatitis B vaccination. Before widespread immunization, hepatitis B posed a significant occupational risk for healthcare and emergency workers, who were frequently exposed to infectious fluids without adequate protective measures. A 10-fold decrease in infections among healthcare workers was observed after the introduction of vaccination in 1983 [9]. Over time, vaccination programs have successfully reduced the incidence of acute VHB,however, chronic cases persist, particularly among older, unvaccinated cohorts. Unlike hepatitis B, no vaccine is currently available for hepatitis C, and the burden of chronic VHC remains high, especially among PWID and incarcerated individuals [22].

The economic burden associated with hepatitis treatment is substantial. Antiviral therapies for chronic hepatitis B and C are costly and require prolonged treatment regimens. In cases of advanced liver disease, complications such as cirrhosis and hepatocellular carcinoma may necessitate invasive and expensive interventions, including liver transplantation and albumin dialysis [29], [18]. The financial strain on healthcare systems underscores the importance of primary prevention strategies, including vaccination, harm reduction programs, and early screening initiatives.

The most effective approach to reducing the incidence of viral hepatitis is prevention. The implementation of universal hepatitis B vaccination in the Czech Republic in 2001 has significantly reduced new infections, particularly in younger age groups [26]. However, the long-term effectiveness of this program at the population level requires continuous evaluation. In contrast, hepatitis C prevention relies heavily on harm reduction interventions, including needle exchange programs, opioid substitution therapy, and improved access to antiviral treatment [4]. Despite advancements in direct-acting antiviral (DAA) therapies, challenges remain in identifying and treating undiagnosed cases, particularly among high-risk populations [13].

This study aims to analyze the incidence of VHB and VHC in Czechia in the post-vaccine era, focusing on epidemiological trends over the last two decades (Table 1). By comparing the incidence of hepatitis B and C, this research seeks to assess the long-term impact of vaccination programs, identify key risk factors influencing disease transmission, and highlight areas requiring targeted public health interventions. Given the persistence of chronic hepatitis cases and the ongoing risk among vulnerable groups, a comprehensive understanding of these trends is essential for guiding future prevention and control strategies.

**Table 1:** Incidence of VHB and VHC in the years 2000-2021 in Czechia (absolute numbers)

**Year**	**VHB**		**VHC**	
	**Acute VHB**	**Chronic VHB**	**Acute VHC**	**Chronic VHC**
2000	604	3	319	318
2001	457	95	276	522
2002	413	122	213	645
2003	370	152	182	664
2004	392	139	197	671
2005	361	211	162	682
2006	307	221	135	887
2007	307	248	138	843
2008	306	173	169	805
2009	247	195	141	702
2010	244	130	114	595
2011	192	159	101	711
2012	154	146	117	677
2013	133	144	134	739
2014	105	192	89	778
2015	89	191	116	840
2016	73	204	123	981
2017	85	245	116	876
2018	54	269	122	928
2019	41	276	103	1035
2020	27	142	88	682
2021	17	127	89	573
**Total**	**3784**	**4978**	**3244**	**16154**

**Fig. 1:**
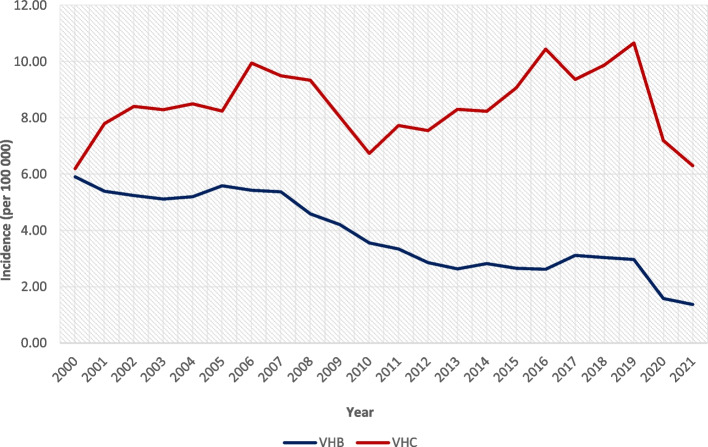
Incidence of VHB and VHC in the years 2000-2021

The Fig. 1 illustrates the incidence of both diseases from 2000 to 2021. Both diseases show a downward trend, but the COVID-19 pandemic in 2020-2021 must be considered. Due to the restrictive measures implemented in the Czech Republic to limit the spread of COVID-19, there has been a subsequent decrease in the number of hepatitis cases.

**Fig. 2:**
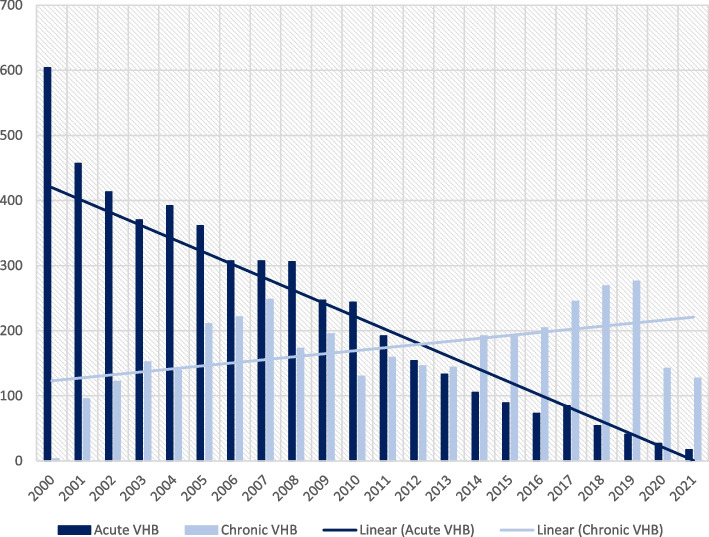
Incidence of acute and chronic VHB in the years 2000-2021

The Fig. 2 illustrates the incidence of acute and chronic forms of VHB from 2000 to 2021 shows that the acute form has been decreasing since 2000, while the chronic form has shown a variable trend. In the last four years, the incidence of both forms has decreased

**Fig. 3:**
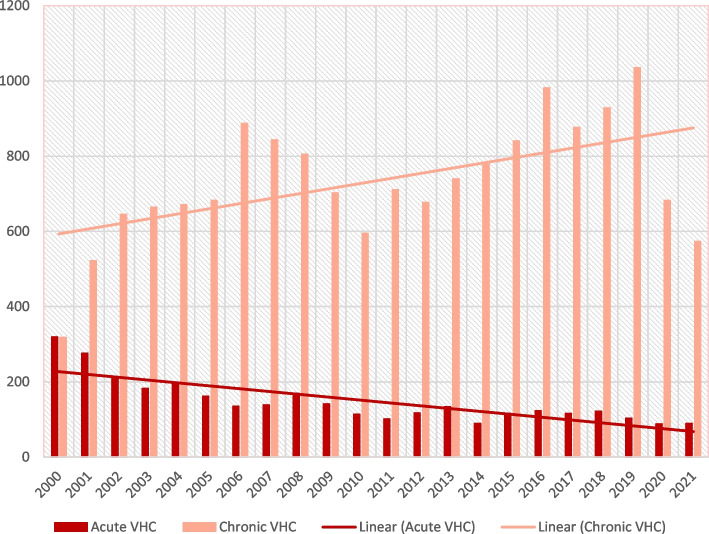
Incidence of acute and chronic VHC in the years 2000-2021

The Fig. 3 illustrates the incidence of acute and chronic forms of HCV in Czechia from 2000 to 2021 shows a decrease in acute cases since 2000. The trend for chronic cases has been variable, but there has been a decrease in the past four years, similar to HBV.

## Methods

### Study design

This study is a retrospective observational analysis aimed at understanding the epidemiological patterns of hepatitis B (VHB) and hepatitis C (VHC) in Czechia over the last two decades (2000–2021). It focuses on identifying incidence trends, analyzing the demographic characteristics of affected populations, and evaluating potential risk factors and transmission routes for these viral infections. Additionally, it compares VHB and VHC incidence in the post-vaccine era to determine the effect of hepatitis B vaccination.

### Data sources

Data for this study were obtained from national infectious disease reporting systems used in Czechia. In Czechia hepatitis B (VHB) and C (VHC) are both diseases reported through reporting systems EPIDAT and since 2018 system ISIN (The Infectious Disease Information System). System ISIN currently ensures mandatory reporting, registration, and comprehensive analysis. ISIN is a vital electronic reporting system used throughout the Czechia to report cases of infectious diseases. Both systems collect epidemiological data that include the patient's demographic details, risk factors, transmission route, and clinical presentation (acute or chronic).

Healthcare providers, who are typically the first to diagnose infectious diseases, have the responsibility of reporting cases to one of the 14 Regional Public Health Authorities. The diagnoses reported in ISIN follow the International Classification of Diseases, 10 th Revision, ensuring standardized and accurate disease reporting. The National Institute of Public Health plays a crucial role in this process by ensuring the accuracy and reliability of the data.

### Study population

Data are collected based on the Commission Implementing Decision (EU) 2018/945 of 22 June 2018 and the national legislation of the Czech Republic, Decree No. 389/2023, where case definition is presented and described. The data used for this analysis are fundamentally the data of confirmed cases based on these regulations. Every individual meeting the case definition criteria was tested in an accredited laboratory. All laboratories in Czechia are accredited according to the standards ČSN EN ISO 15189 ED. 3:2023 or ČSN EN ISO 15189 ED. 2:2013, which outline the requirements for quality and competence in healthcare laboratories.

Once a case is confirmed based on these parameters, it is reported by all laboratories and healthcare providers in accordance with the regional decree regulating epidemiological surveillance, to the respective regional public health authority. The public health authority records the cases according to strict standardized criteria.

Testing procedures and definition of the acute and chronic form for hepatitis B and C follows the Diagnosis and Therapy of hepatitis B infection - Czech national guidelines [10] and Standard diagnostic and therapeutic approach to chronic hepatitis C virus (HCV) infection 22 - guidelines [30].

The study included all reported cases of VHB and VHC from 2000 to 2021. In total, 28,160 cases were analyzed, of which 8,762 were VHB and 19,398 were VHC. This dataset was anonymized, ensuring compliance with privacy and ethical standards, and included key information about year of diagnosis, geographic region (district-level data), gender and age group, risk behaviours (e.g., intravenous drug use, sexual practices), transmission routes (e.g., parenteral, sexual, vertical transmission).

### Data management

Certain data exhibited inconsistencies, indicating insufficient coordination among specialists during reporting. Furthermore, the data on the incidence of acute VHC might be impacted by the inadvertent inclusion of chronic VHC cases, potentially leading to the misreporting of chronic VHC cases as acute. The European Centre for Disease Prevention and Control (ECDC) also highlights concerns about the inconsistency and validity of data related to reporting viral hepatitis. Consequently, there is a pressing need for the enhancement of systems designed for data collection in European countries, as emphasized by the ECDC (Prevention of hepatitis B and C in the EU/EEA 2022).

The obtained data were unified and cleaned. Cases that appeared multiple times due to multiple reports were identified and removed. Cases with missing or inconsistent information regarding transmission routes and risk factors were carefully reviewed. Where feasible, data were filled using additional information provided in the notes section of the reporting system.

This research utilized anonymized data from public health reporting systems, so individual patient consent was not required. The study followed the ethical guidelines for epidemiological research established by the National Institute of Public Health and complied with the General Data Protection Regulation (GDPR) for data security and confidentiality.

### Data analysis

The study used descriptive statistics and spatial visualization to identify trends and regions or demographic groups with higher infection rates. Incidence rates per 100,000 inhabitants were calculated annually, categorized by region, gender, age, and clinical manifestation (acute vs. chronic). These rates were plotted over time to observe trends in VHB and VHC infections over 20 years. Gender distribution and age-specific incidence were analyzed to identify the most affected groups. The impact of VHB vaccination on younger populations was also examined.

The study focused on risk groups, especially intravenous drug users (IDUs), who were overrepresented in VHB and VHC cases. The proportion of cases related to IUD was monitored over time and across regions, with spatial visualization pinpointing potential transmission hotspots. Additional risk factors, such as occupational exposure in healthcare and cosmetic services, were also investigated based on reported VHB cases linked to these activities.

Using district-level data, a spatial visualization was conducted to show the distribution of VHB and VHC across different regions of Czechia. Incidence maps for the years 2000, 2010, and 2020 were created to pinpoint areas with consistently high infection rates and regions where interventions might have been particularly effective. A time-series analysis was performed to assess trends in the incidence of both VHB and VHC. This included evaluating the impact of the universal VHB vaccination program introduced for infants in 2001, as well as the potential impact of the COVID-19 pandemic. The COVID-19 pandemic has significantly impacted all areas of public health. During this period, the number of reported cases of not only hepatitis but also other infectious diseases decreased. This decline was primarily due to the restrictive measures implemented in the Czech Republic at that time, including travel restrictions, limitations on social interactions, school and business closures, restrictions on the movement of people, and the closure of public spaces. These measures significantly contributed to the reduction in the number of cases.

The transmission routes for VHB and VHC were analyzed. Infection routes (e.g., sexual transmission, needle-sharing) were identified where possible. Cases with undetermined routes, especially for VHC, were reviewed with supplementary data. A sub-analysis explored healthcare-associated infections in medical or dental settings and among healthcare workers. Special attention was given to imported hepatitis cases, particularly from high-prevalence regions like Ukraine and Vietnam, to assess the impact of migration on VHB and VHC epidemiology in Czechia.

## Results

### Hepatitis B

#### Vaccination

The hepatitis B infection rate in Czechia was alarmingly high before the vaccine, with about 27 cases per 100,000 people. In 1982, healthcare workers had an even higher incidence, between 177 and 2,083 cases per 100,000 [9]. The first evidence supporting the efficacy and safety of the hepatitis B vaccine emerged in the early 1980 s [27], [25], [17]. Subsequently, vaccinating vulnerable individuals became a public health priority. In 1983, Czechia mandated the first compulsory vaccination for hospital medical staff. This led to a 10-fold decrease in infections among health workers, reducing the rate to 17 cases per 100,000, which was lower than the general population rate.

Individuals born after 1989 should be protected against VHB. In Czechia, a vaccination program for infants and unvaccinated children over 12 years was started in 2001, with coverage ranging from 84% to 94% [26]. Currently, Decree No. 355/2017 requires children over 9 weeks to receive a hexavalent vaccine for six diseases, including HBV, in three doses. It also requires vaccination for individuals in dialysis programs and those awaiting organ transplants. Specialized vaccinations are given to those at elevated risk. Employees and members of the integrated rescue system receive a combined vaccine for hepatitis A and B. Special vaccinations are not given to those with a documented history of illness or an antibody titer over 10 IU/l. However, individuals not in the special vaccination category can opt for vaccination, including travellers to high-prevalence regions [24], [26].

#### Epidemiology in Czechia

The socio-demographic analysis indicates that VHB is more common among men (63.9%) than women (36.1%). The age groups most affected are 20–29 years (26.8%) and 30–39 years (22.7%). The average age at the time of reporting varies based on the clinical manifestation of the disease. The acute form is usually diagnosed at a younger age than the chronic form, likely due to asymptomatic cases of the acute variant. Many chronic form cases were discovered incidentally during routine examinations such as pre-operative or pregnancy screenings. In Czechia, pregnancy screening is conducted in the 14 th week of pregnancy. A total of 80 cases were diagnosed during pregnancy screening, highlighting the importance of regular screening examinations.

During the observation period, the highest number of cases was consistently reported in Prague (22.1%), followed by Středočeský (15.5%), Ústecký (13.3%), and Moravskoslezský (9.6%) regions. The Vysočina region consistently reported the fewest cases.

**Fig. 4:**
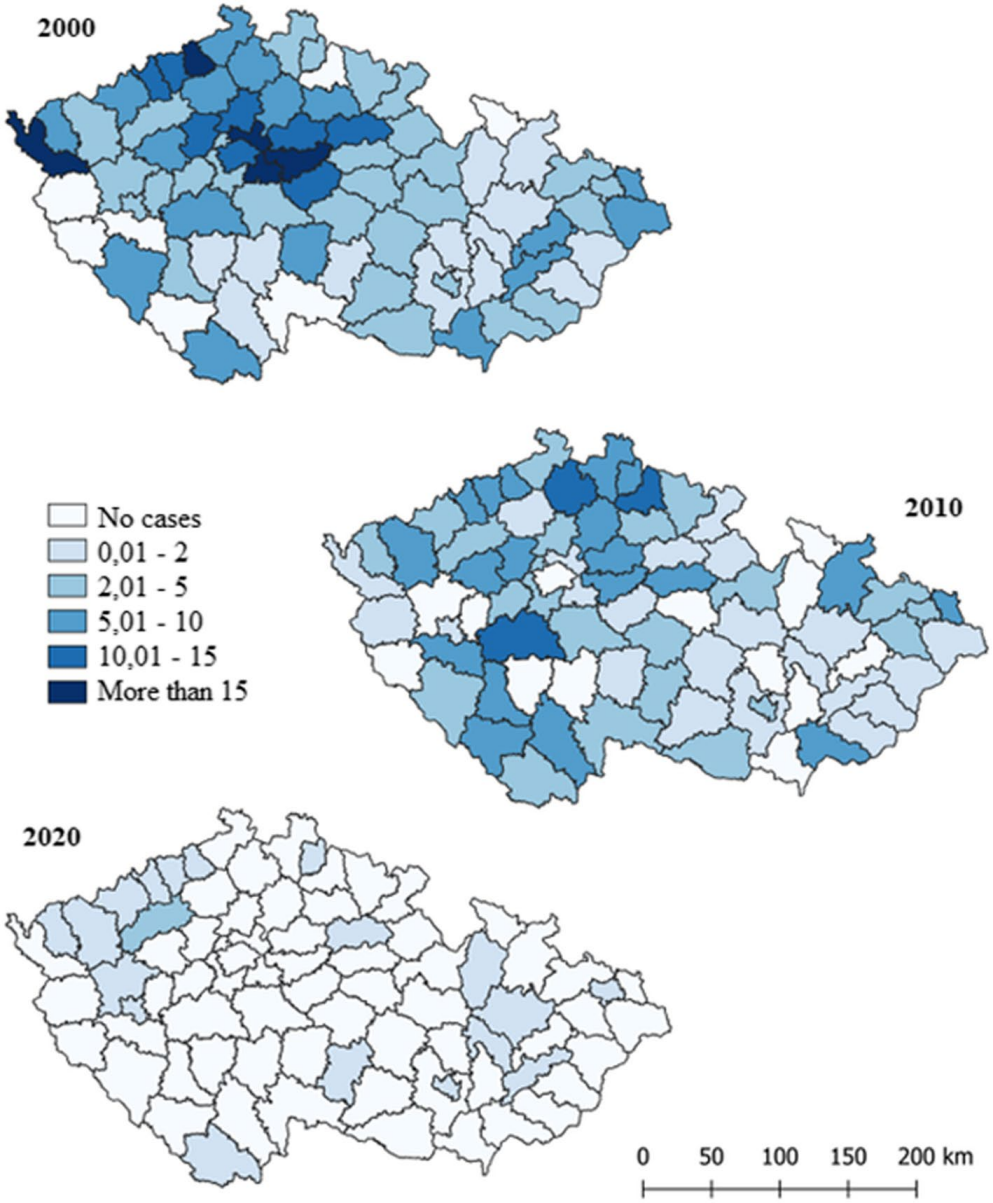
Spatial visualization of the incidence of VHB per 100,000 inhabitants by Czech districts for the years 2000, 2010, and 2020

Spatial visualization in Figure 4 shows disease incidence per 100,000 inhabitants by districts for 2000, 2010, and 2020. In 2000, Prague-Východ, Kolín, Cheb, and Ústí nad Labem had the highest cases with 607 reported. By 2010, cases decreased to 374, with Příbram, Semily, and Česká Lípa being the most affected. In 2020, only 167 cases were reported, with Louny district having the highest incidence.

The incidence of acute VHB has consistently decreased due to vaccination efforts since 2001, while the chronic form has shown variable trends, peaking in 2005 and decreasing sharply by 2021.

#### Risk groups

Risky behavior was identified in 29.5% of cases among those infected. The main risk groups were IDUs at 53.9%, men who have sex with men individuals at 7.6%, and promiscuous individuals at 6.9%. Methamphetamine was the most frequently used drug among those infected with VHB, accounting for 55%, followed by heroin at 29%, or a combination of both at 28%. Other substances used included MDMA, LSD, and THC (rare).

**Fig. 5:**
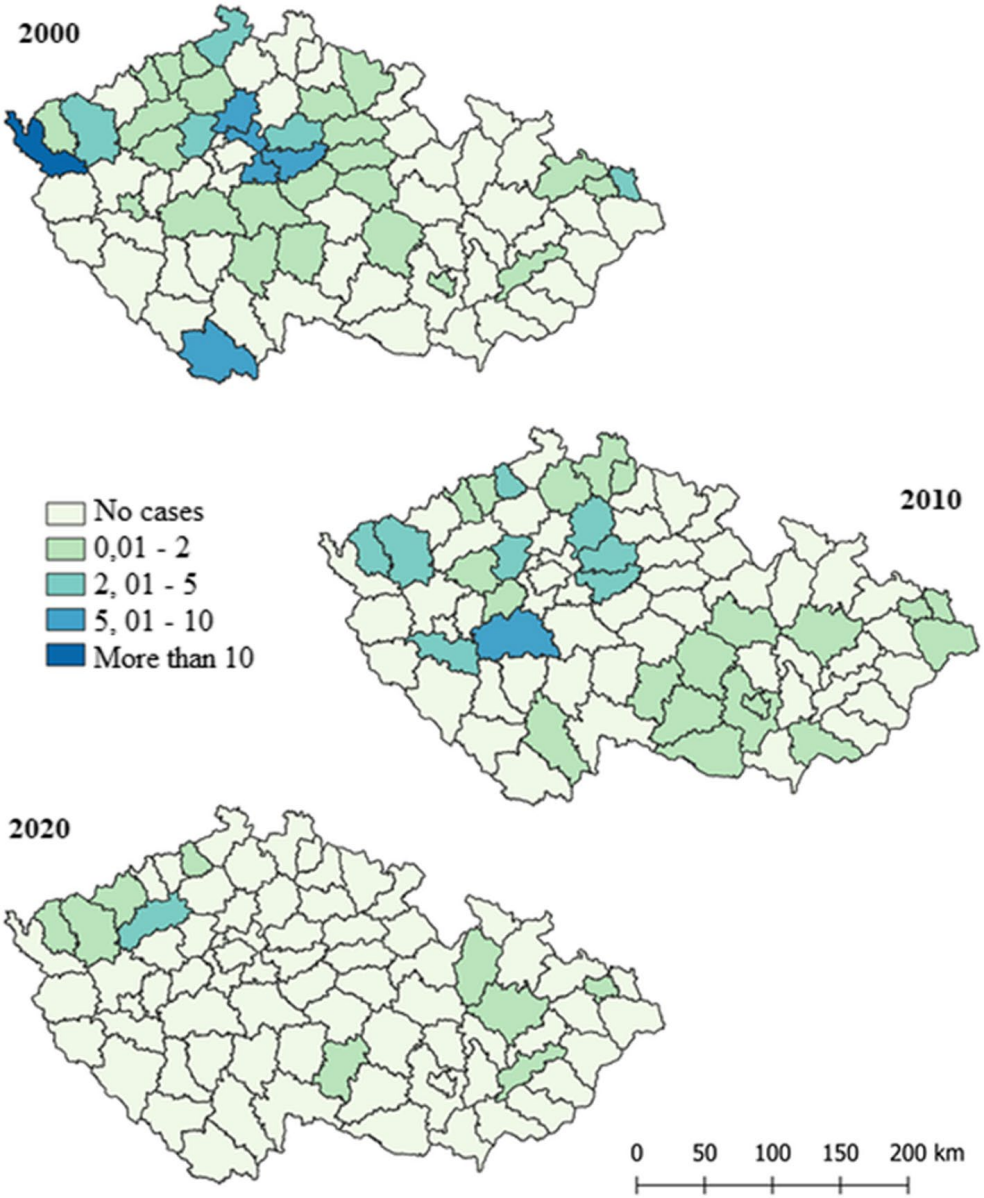
Spatial visualization of the incidence of IDUs among VHB patients per 100,000 inhabitants by Czech districts for the years 2000, 2010 and 2020

Figure 5 shows the incidence of IDUs among VHB patients in 2000, 2010, and 2020. There is a significant correlation between the overall population and IDUs in specific districts. In 2000, with 158 cases, the highest representation was in Cheb, followed by Mělník, Praha-Východ, Kolín, and Český Krumlov. By 2010, with 75 cases, notable districts were Příbram, Plzeň-Jih, Kolín, Nymburk, Mladá Boleslav, Kladno, Sokolov, Karlovy Vary, and Ústí nad Labem. In 2020, only 12 cases were reported, with the highest in Louny district.

#### Transmission

The transmission route was undetermined in 68% of cases. The most common transmission route is parenteral outside healthcare facilities (10%). Sharing infected needles among IDUs is a frequent cause. Transmission also occurs through amateur tattooing or piercing at home without sterile instruments. Some cases resulted from injuries by infected needles in outdoor settings (playgrounds, public transport, etc.).

Professional infections through services like hairdressing, manicure, pedicure, piercing, or tattooing were reported in several cases. The second most common transmission route was contact with an infected individual or HBsAg carrier. Risky unprotected sexual intercourse, often during overseas holidays or sexual services, was frequently cited in the details of the reports. Infections within families were reported due to sharing hygiene items, close contact, or perinatal transmission. Parenteral infections in medical facilities were infrequent (needle stick injuries, transfusions, surgical procedures, etc.). A total of 462 imported disease cases were reported, with Vietnam (21%), Ukraine (20%), and Slovakia (7%) being the most common sources. The number of foreigners legally residing in the Czech Republic continued to grow until 2021 (Table 2) [3].

**Table 2:** The approx. umber of foreigners legally residing in the Czech Republic (in thousands)

**Year**	**Approx. Number of Foreigners**
2000	200 thousand
2005	280 thousand
2010	425 thousand
2015	465 thousand
2020	630 thousand
2021	660 thousand

### Hepatitis C

#### Epidemiology in Czechia

The socio-demographic analysis shows a higher prevalence of VHC among men (67%) than women (33%). The most represented age group is 20–29 years (38%), followed by 30–39 years (28%). The research highlights that the most represented age group is between 20 and 34 years. The average age at reporting varies by the clinical form of the disease, with acute cases diagnosed younger than chronic cases, likely due to asymptomatic acute cases.

**Fig. 6:**
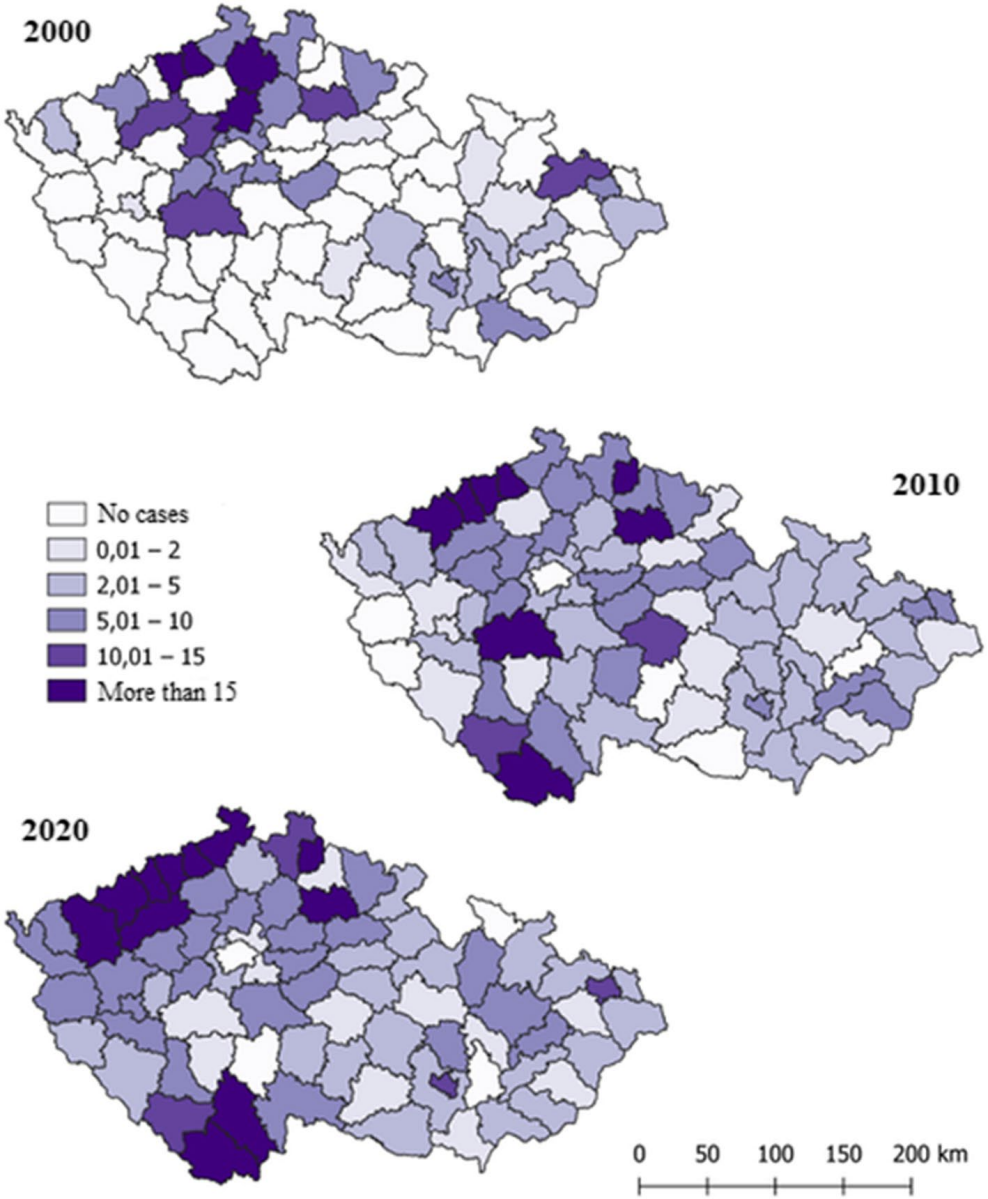
Spatial visualization of the incidence of VHC per 100,000 inhabitants by Czech districts for the years 2000, 2010 and 2020

Many chronic cases were found during routine exams, such as pre-operative checks or before serving a sentence. The highest number of cases were in Prague (20.1%), followed by Ústecký (17.2%), Středočeský (10.4%), and Jihočeský (10.4%). Figure 6 shows the disease's incidence per 100,000 inhabitants by district in 2000, 2010, and 2020. In 2000, with 637 cases, the most affected districts were Teplice, Ústí nad Labem, Česká Lípa, Mělník, Příbram, Jičín, and Opava. By 2010, with 709 cases, the most affected districts were Chomutov, Most, Teplice, Ústí nad Labem, Jablonec nad Nisou, Jičín, Příbram, and Český Krumlov. In 2020, with 770 cases, the highest incidence was in Karlovy Vary, Chomutov, Louny, Most, Teplice, Ústí nad Labem, Děčín, Jablonec nad Nisou, Jičín, České Budějovice, and Český Krumlov.

The incidence trends show differences between the acute and chronic forms of the disease. The acute form of VHC has been decreasing over time, while the chronic form has shown variable trends. The highest number of cases was in 2019, with an incidence of 10.67 per 100,000 inhabitants. Since then, the incidence has decreased to 6.30 in 2021.

#### Risk groups

Risky behaviour was documented in 66.9% of cases. The most prevalent risk group was IDUs (86%), followed by individuals with antisocial behaviour engaging in intravenous drug use (2.24%) and those with antisocial behaviour alone (2.03%). The predominant drug used among individuals infected with HCV was methamphetamine (65%), followed by heroin (20%) and a combination of both drugs (15%). Other commonly used substances included MDMA, LSD, THC, intranasal use of pervitin and cocaine, and inhalation of toluene.

**Fig. 7:**
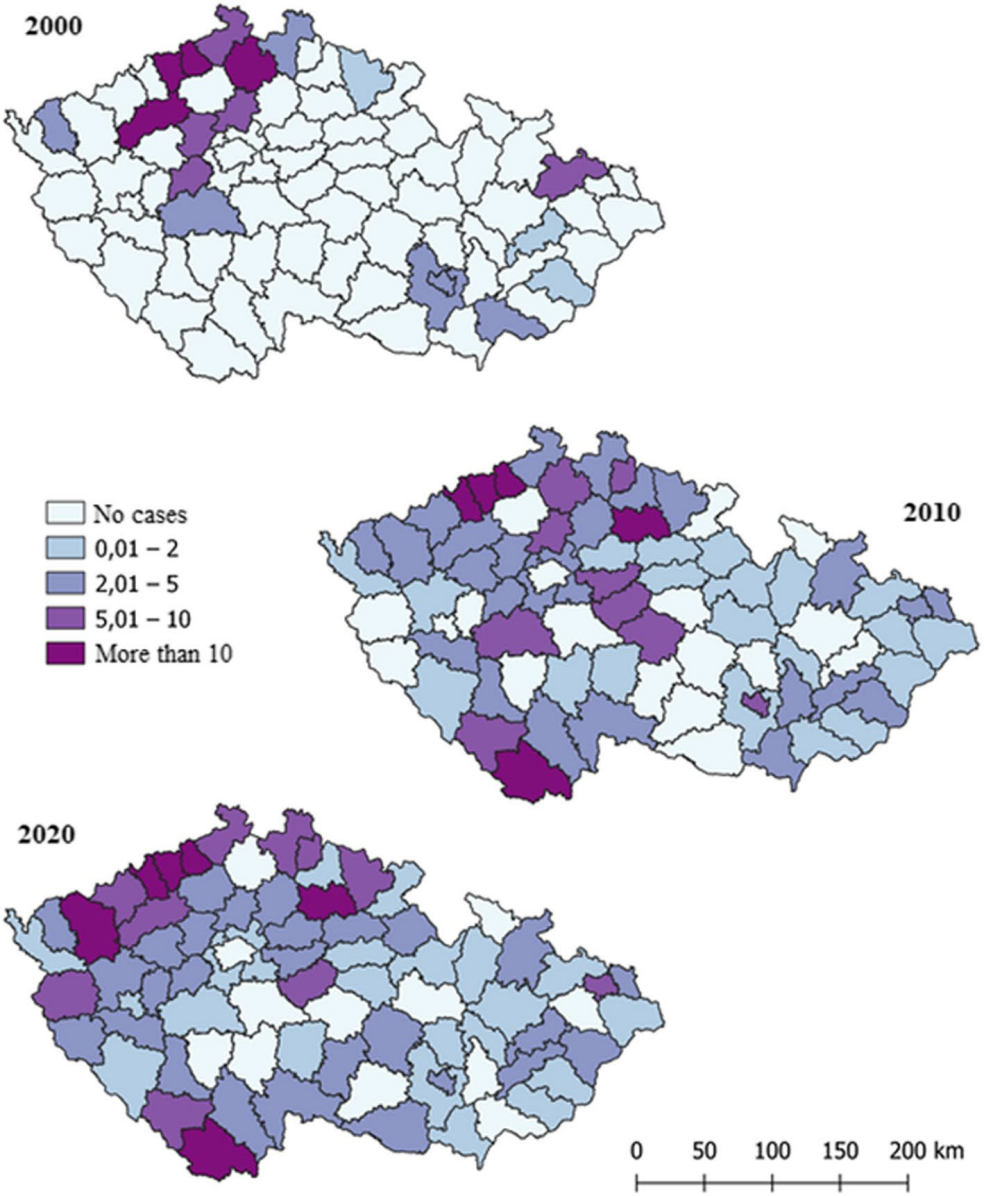
Spatial visualization of the incidence of IDUs among VHC patients per 100,000 inhabitants by Czech districts in 2000, 2010, and 2020

Figure 7 shows the representation of IDUs with VHC in 2000, 2010, and 2020. The incidence rates in both the overall surveyed population and among IDUs in specific districts show a significant correlation. In 2000, with 348 reported cases, the most represented districts were Louny, Teplice, Ústí nad Labem, and Česká Lípa. By 2010, with a total of 410 cases, the districts of Most, Teplice, Ústí nad Labem, Jičín, and Český Krumlov were most represented. In 2020, 373 cases were reported, with the highest representation in the districts of Karlovy Vary, Most, Teplice, Ústí nad Labem, Jičín, and Český Krumlov.

#### Transmission

In more than half of the cases (53.9%), the transmission route is unknown. The most common identified route is parenteral administration outside healthcare facilities (25.9%). Reports indicate that the main cause is infected needles among IDUs. Transmission also occurs through amateur tattoos or piercings done at home without sterile instruments.

Unlike VHB infection, transmission through unprotected sexual intercourse was rarely reported and typically involved sexual contact with a partner within the IDU group. A relatively large group consisted of individuals serving sentences, who were reported to have both clinical forms. During their sentences, transmission occurred through amateur tattoos or piercings and the sharing of hygiene tools (mostly razors). In isolated cases, transmission happened through sexual contact, including incidents of rape between fellow prisoners in cells. Within the units, infection occurred through injuries with infectious needles in outdoor environments or through sexual assault. Parenteral infection in medical facilities (needle stick injuries, transfusions, surgical procedures, etc.) was infrequent and often related to procedures performed abroad. A total of 298 cases of imported diseases were reported during the monitored period. The countries with the highest frequency of imports were Ukraine (30%), Slovakia (10%), and Georgia (5%). The number of foreigners legally residing in the Czech Republic continued to grow until 2021.

## Discussion

Despite the high contagiousness of the hepatitis B virus, there are significantly fewer cases of hepatitis B compared to hepatitis C, for which no vaccination exists. Over the past 21 years, from 2000 to 2021, the number of hepatitis C infections has been 2.2 times higher than hepatitis B infections in Czechia. Specifically, over these 20 years, only 8,762 cases of hepatitis B virus and 19,398 cases of hepatitis C virus were recorded. This distribution is primarily due to the hepatitis B vaccination. Referring to Figure No. 4, we can observe a rapid decline in the number of hepatitis B infections every decade. For instance, from 2000 to 2010, the number of detected cases decreased by 1.62 times (2000 - 607 cases, 2010 - 374 cases). Furthermore, between 2010 and 2020, the number of cases decreased even more significantly, by a factor of 2.23 (2020 - 167 cases). In contrast, the incidence of hepatitis C has not decreased over the past 20 years and has even slightly increased. In 2000, there were 637 cases, in 2010 - 709 cases, and in 2020 - 770 cases.

The significant drop in hepatitis B cases reported in 2020 can be linked to the COVID-19 pandemic and the reduced pursuit of inpatient and outpatient care. This is further evidenced by the decrease in hepatitis C incidence from 2019 to 2021, with rates falling from 10.67 in 2019 to 6.3 in 2021. The pandemic has globally limited access to infectious hepatitis testing programs. According to a survey by the World Hepatitis Alliance (WHA) conducted in 32 countries between March 30 and May 4, 2020, only 47 out of 132 respondents (36%) indicated that people could freely access testing services [28]. Additionally, the demand for health services dropped due to movement restrictions and people's fears of contracting COVID-19 [13, 22]. The COVID-19 pandemic may have also impacted the hepatitis B vaccination program. Evidence suggests that routine immunization and vaccination programs are often the first to be affected by epidemics, political instability, or economic crises [22].

Men are more likely to have viral hepatitis (B and C) than women, possibly due to higher engagement in risky behaviours like drug use [16], [2]. Among those with hepatitis B, 53.9% used injected drugs, while 86% of hepatitis C cases involved injecting drug users. Additionally, 7.6% of risky hepatitis B cases were among men who have sex with men. More educational campaigns for at-risk groups are recommended.

Research shows that VHB is more common among men, especially those aged 20–29, with the highest number of cases in Prague, the Central Bohemian Region, and the Ústí Region. Although the incidence has decreased over the past twenty years, Northern Bohemia remains the most affected area due to lower socio-economic status and higher drug use, particularly among IDUs. Preventive measures, including healthcare worker vaccinations in 1983 and compulsory child vaccinations since 2001, have reduced the incidence in the Czech Republic, with a vaccination rate over 95% by 2020. Similarly, VHC is prevalent among men aged 20–29, with most cases in Prague, the Ústí Region, and the Central Bohemian Region. Northern Bohemia districts are heavily affected due to low socioeconomic status and high drug use. The incidence of VHC has shown slight changes since 2000 but remains persistent due to limited prevention and no effective vaccine, emphasizing the need for non-specific prevention.

In both diseases, determining the route of transmission was not possible in more than half of the cases. The most frequently reported transmission route was parenteral transmission outside a healthcare facility. For VHB, transmission mainly occurs during intravenous drug use or risky sexual intercourse. The most at-risk group includes IDUs, followed by men who have sex with men and promiscuous individuals. With VHC, sexual contact transmission is less frequent, and IDUs are clearly the most at-risk group. A relatively large number of VHC cases have been recorded in prisons, where infection occurred through shared razors or amateur tattooing and piercing. For both diseases, there have been reported cases of infection through amateur tattooing or piercing, in services like hairdressing, manicure, pedicure, or through needlestick injuries outdoors. A small percentage were imported cases. Despite this, it is important to increase society's awareness about travel medicine and the possibility of vaccination before traveling to countries with a high prevalence.

Cosmetic services have emerged as a factor influencing the incidence of hepatitis B [11, 12]. Some hepatitis B cases in Czechia were linked to occupational infections outside medical institutions, such as in nail salons and tattoo parlours [8]. The cosmetic industry is poorly regulated, and both beauticians and their clients often lack awareness of basic care standards. A study in São Paulo, Brazil, revealed a low level of knowledge about hepatitis B and C transmission among cosmetic workers, with 93 percent of those surveyed not pre-treating their instruments, and 8 percent having hepatitis B and 2 percent hepatitis C [21]. Given that the hepatitis B virus can be transmitted through untreated manicure scissors, even without direct contact with the blood of hepatitis B carriers [14], the need for briefings and awareness campaigns for beauty salon workers, as well as the introduction of mandatory vaccination for all employees who might come into contact with the blood or other biological material of their clients, becomes evident.

Another factor affecting the incidence of hepatitis B is population migration, which becomes particularly relevant during armed conflicts and the resettlement of large groups of people. Cases of viral hepatitis B were often imported, totalling 462 cases. The leading exporting countries were Vietnam (21%), Ukraine (20%), and Slovakia. Consequently, due to constant population migration, even in countries relatively free of hepatitis B and C, significant statistics may emerge as the hepatitis B and C epidemiology of these countries begins to be influenced by migratory flows [1, 6]. Additionally, migrants may be unaware of their illness and may not visit a physician due to lack of insurance, posing potential risks to their partners or children. Vietnam has a high incidence of hepatitis B and its complications: the country had the sixth highest incidence of hepatocellular carcinoma in the world and the third highest liver cancer mortality rate in the world in 2018 [19]. Furthermore, it is estimated that 8.4 million people in Vietnam are living with chronic hepatitis B virus infections. In many regions of Vietnam, the prevalence of HBsAg in the population ranges from 10% to 20% [20]. Ukraine, in turn, has the second highest incidence of hepatitis B among countries in Central and Eastern Europe and Central Asia [15]. This is due to low vaccination coverage, including the hepatitis B vaccine, since 2009. The economic crisis, prolonged political instability, and a protracted military conflict with Russia have led to a deterioration of the socio-economic situation and the internal and external migration of more than one million people [23]. This has created additional obstacles to an already weakened national immunization program: as of 2019, only 76% of the child population had received three doses of the hepatitis B vaccine. In 2011, vaccination coverage was at a historic low of 21%. Vaccination rates may be even lower after 2022. Consequently, Czechia's VHB statistics may change dramatically, as Ukrainians, whose immunization program has been flawed for the past 15 years, now dominate the mix of foreign nationals living in Czechia. Provision should be made for testing refugees and other foreign nationals for viral hepatitis B and C, and the possibility of unscheduled vaccination for both adults and children should be available.

Infectious hepatitis B and C are prevalent diseases in Czechia. However, hepatitis B has been managed effectively due to a well-established immunization program, which includes universal immunization for children and targeted immunization for at-risk adults. The number of confirmed hepatitis B cases is steadily declining each year. Conversely, the number of hepatitis C cases has not decreased over the years, and the disease remains poorly prevented and controlled.

To prevent hepatitis, it is recommended to inform at-risk groups and conduct awareness campaigns for beauty workers. Given the high number of IDUs among the infected, there should be a focus on non-specific prevention and anti-drug policies. Harm reduction programs, opioid substitution therapy (OST), and sexual health programs are crucial tools. Additionally, strengthening health support at the community level is necessary. It would also be wise to recommend mandatory vaccination not only for healthcare workers but for all employees of beauty salons and tattoo parlours who come into contact with blood or other biological materials from clients. Furthermore, considering recent global events, free hepatitis B vaccination should be provided for refugees, both adults and children, along with additional testing for viral hepatitis in this group.

Despite significant progress in recent years due to effective prevention and treatment, there are still challenges in eliminating VHB and especially VHC. Finding solutions for nationwide elimination programs is crucial to meet the 2030 targets. Accurate monitoring of epidemiological trends and identifying areas needing intervention are essential.

By leveraging new insights gained from this meticulous analysis, we can design targeted prevention strategies that address the unique challenges and vulnerabilities within these risk groups. These strategies would include enhanced screening programs, public awareness campaigns, and educational efforts aimed at both healthcare professionals and the general population.

Moreover, the integration of innovative treatment options and improved access to healthcare services will be crucial. Ensuring that high-risk individuals receive timely and appropriate medical care can significantly mitigate the spread of these viruses. This multi-faceted approach would not only focus on preventing new infections but also on providing comprehensive care and support to those already affected.

The limitations of this study highlight key challenges in accurately assessing the epidemiology of hepatitis B and C in Czechia. Underreporting, especially of asymptomatic cases, may have led to an underestimation of the true disease burden, affecting the reliability of incidence trends. Additionally, the COVID-19 pandemic likely disrupted healthcare access and infectious disease surveillance, resulting in a temporary decline in reported cases rather than an actual reduction in infections. The lack of detailed behavioral and demographic data further limits the ability to identify specific risk factors and transmission dynamics, which are essential for targeted prevention efforts. Migration has been identified as a contributing factor to hepatitis B and C cases; however, the absence of individual-level data, such as vaccination status and length of stay in Czechia, prevents a more precise evaluation of its impact.

These limitations underscore the need for improved surveillance systems, better data collection on risk behaviors and migration history, and expanded screening programs to ensure timely identification and intervention, particularly among high-risk and migrant populations. Strengthening these areas would enhance the ability to develop and implement more effective prevention and control strategies.Existing programs, while relatively effective, need to be expanded and adapted to meet the evolving needs of patients. This means incorporating up-to-date scientific knowledge and clinical practices into national health policies. By addressing the current demands and considering the future landscape of viral hepatitis management, we can create a more resilient healthcare system.

## Conclusions

This study analyzed the epidemiology of hepatitis B (VHB) and hepatitis C (VHC) in Czechia from 2000 to 2021, focusing on incidence trends, risk groups, and transmission routes. Our findings highlight the significant impact of vaccination on reducing VHB incidence, while VHC remains a persistent public health concern, primarily due to the lack of a vaccine and ongoing transmission among intravenous drug users (IDUs).

The analysis revealed that VHC cases outnumbered VHB cases by a factor of 2.2, with 19,398 reported VHC infections compared to 8,762 VHB cases over the study period. While VHB incidence has significantly declined due to the introduction of universal infant vaccination in 2001, VHC trends have remained variable, with a peak incidence in 2019 followed by a decline, likely influenced by the COVID-19 pandemic and associated healthcare disruptions. The highest incidence rates for both infections were observed in Prague and the Ústecký region, areas with a higher concentration of at-risk populations, particularly IDUs.

The study confirmed that the primary mode of transmission for both infections is parenteral exposure outside healthcare settings, with IDUs being the most affected group. Among VHB cases with known risk factors, 53.9% were IDUs, and 7.6% were men who have sex with men (MSM). In VHC cases, IDUs accounted for 86% of infections, with additional risks identified among incarcerated individuals due to shared razors and amateur tattooing or piercing. The data also suggest a role for non-medical cosmetic services (e.g., manicures, pedicures, and hairdressing) in VHB transmission, underlining the need for improved hygiene standards and mandatory vaccination of workers in these industries.

Migration has emerged as another key factor influencing hepatitis epidemiology in Czechia. The study identified 462 imported VHB cases, with the highest proportions originating from Vietnam (21%) and Ukraine (20%). Given the high prevalence of chronic hepatitis B in these regions, targeted screening and vaccination programs for migrants should be prioritized.

To achieve the World Health Organization’s goal of eliminating viral hepatitis by 2030, continued investment in prevention, harm reduction, and treatment programs is essential. For VHB, increasing vaccine uptake among at-risk adult populations, particularly healthcare and cosmetic industry workers, should be a priority. For VHC, expanding harm reduction strategies such as opioid substitution therapy (OST), needle exchange programs, and prison-based healthcare interventions is crucial. Additionally, improved surveillance and data quality, particularly in identifying transmission routes, will enhance the effectiveness of public health responses.

Despite progress in reducing the burden of viral hepatitis in Czechia, particularly for VHB, further efforts are needed to address the persistent challenges associated with VHC transmission. Strengthening multi-sectoral collaboration and integrating innovative screening and treatment strategies will be essential in working towards the effective control and eventual elimination of these infections.

### Study limitations

This study is limited by potential underreporting in national infectious disease reporting systems, particularly for asymptomatic cases. The COVID-19 pandemic likely influenced case reporting in 2020–2021 due to reduced healthcare access. The lack of detailed behavioral and demographic data, may have impacted incidence trends. While migration was identified as a contributing factor, individual-level data such as vaccination status and length of stay in Czechia were unavailable. Despite these limitations, the study provides valuable insights into hepatitis epidemiology and underscores the need for improved surveillance, targeted screening, and enhanced prevention efforts, particularly among high-risk and migrant populations.

## Data Availability

Data is provided within the manuscript.
